# Chronic Metritis—Prof. Scanzoni’s Treatment

**Published:** 1867-09

**Authors:** 


### CHRONIC METRITIS—PROF. SCANZONI'S TREATMENT.

Prof. Scanzoni, of Wurtzburg, has never obtained any good effects from anything but the iodide of potassium, and the iodochloride of mercury in direct application to the uterine and vaginal mucous membranes.

He uses, for instance, a liniment containing one drachm of iodide of potassium to one ounce of glycerine, and places every night in the vagina a sponge impregnated with this fluid. The sponge is removed in the morning. This, he says, is the only method of iodine dressing which has ever been found capable of reducing in the course of two or three weeks the size and induration of the inferior segment of the womb, and is infinitely preferable to the application of tincture of iodine and of iodized liniments to the inguinal regions.

Scanzoni has more recently had recourse in the same manner to the introduction into the vagina of the following pomade:

Hydrarg. iodo-chloridi, gr. v.
Adipis, ℥j.

After each application of the remedy which requires the assistance of the speculum, the patient should keep her bed for six or eight hours.

The sponge may then be extracted, and an injection of tepid water should be performed. The epithelium is in general destroyed in the parts which have come into contact with the ointment; exudation follows, and marked decrease of size of cervix. The application may be repeated several times, if necessary, at intervals of ten days or a fortnight.

Scanzoni has completely relinquished the practice of applying tincture of iodine to the vagina or cervix. When excoriations are present, he prefers to all other local remedies rectified pyroligneous acid, pure or mixed with equal parts of creasote. He leaves these modifiers in contact with the ulcerated surfaces, until the sanguineous oozing has ceased, and until the part which is in general of a bright red, has required a dead white color.—*Journal of Practical Medicine and Surgery.*

